# Paternal obesity is associated with *IGF2 *hypomethylation in newborns: results from a Newborn Epigenetics Study (NEST) cohort

**DOI:** 10.1186/1741-7015-11-29

**Published:** 2013-02-06

**Authors:** Adelheid Soubry, Joellen M Schildkraut, Amy Murtha, Frances Wang, Zhiqing Huang, Autumn Bernal, Joanne Kurtzberg, Randy L Jirtle, Susan K Murphy, Cathrine Hoyo

**Affiliations:** 1 Duke Cancer Institute, Duke University Medical Center 2715, Durham, NC 27710, USA; 2 Department of Community and Family Medicine, Duke University Medical Center 104006, Durham, NC 27710, USA; 3 Division of Maternal and Fetal Medicine, Department of Obstetrics and Gynecology, Duke University Medical Center, 4022 Hospital South, Durham, NC 27705, USA; 4 Department of Obstetrics and Gynecology, Division of Gynecologic Oncology, Duke University Medical Center 91012, Durham, NC 27708, USA; 5 Department of Radiation Oncology, Duke University Medical Center 3433, Durham, NC 27710, USA; 6 Department of Pediatrics, Duke University Medical Center 3350, Durham, NC 27710, USA; 7 Department of Obstetrics and Gynecology, Division of Clinical Epidemiology, Duke University Medical Center 2914, Durham, NC 27710, USA

**Keywords:** Epigenetics, DNA methylation, IGF2, obesity, offspring, Newborn Epigenetics Study, Epidemiology

## Abstract

**Background:**

Data from epidemiological and animal model studies suggest that nutrition during pregnancy may affect the health status of subsequent generations. These transgenerational effects are now being explained by disruptions at the level of the epigenetic machinery. Besides *in vitro *environmental exposures, the possible impact on the reprogramming of methylation profiles at imprinted genes at a much earlier time point, such as during spermatogenesis or oogenesis, has not previously been considered. In this study, our aim was to determine associations between preconceptional obesity and DNA methylation profiles in the offspring, particularly at the differentially methylated regions (DMRs) of the imprinted *Insulin-like Growth Factor 2 *(*IGF2*) gene.

**Methods:**

We examined DNA from umbilical cord blood leukocytes from 79 newborns, born between July 2005 and November 2006 at Duke University Hospital, Durham, NC. Their mothers participated in the Newborn Epigenetics Study (NEST) during pregnancy. Parental characteristics were obtained via standardized questionnaires and medical records. DNA methylation patterns at two DMRs were analyzed by bisulfite pyrosequencing; one DMR upstream of *IGF2 *(*IGF2 *DMR), and one DMR upstream of the neighboring *H19 *gene (*H19 *DMR). Multiple regression models were used to determine potential associations between the offspring's DNA methylation patterns and parental obesity before conception. Obesity was defined as body mass index (BMI) ≥30 kg/m^2^.

**Results:**

Hypomethylation at the *IGF2 *DMR was associated with paternal obesity. Even after adjusting for several maternal and newborn characteristics, we observed a persistent inverse association between DNA methylation in the offspring and paternal obesity (β-coefficient was -5.28, *P *= 0.003). At the *H19 *DMR, no significant associations were detected between methylation patterns and paternal obesity. Our data suggest an increase in DNA methylation at the *IGF2 *and *H19 *DMRs among newborns from obese mothers, but a larger study is warranted to further explore the potential effects of maternal obesity or lifestyle on the offspring's epigenome.

**Conclusions:**

While our small sample size is limited, our data indicate a preconceptional impact of paternal obesity on the reprogramming of imprint marks during spermatogenesis. Given the biological importance of imprinting fidelity, our study provides evidence for transgenerational effects of paternal obesity that may influence the offspring's future health status.

## Background

Environmental exposures acquired early in life have been correlated with persistent modifications of the epigenome. The epigenetic information in human cells is stored via mitotically heritable DNA methylation, organization of the chromatin structure (for example, histone modification), and regulatory RNAs. Together, these mechanisms are responsible for regulating gene expression during cellular differentiation during embryonic development and throughout life [1]. Our study focuses on the DNA methylation patterns of the imprinted *Insulin-Like Growth Factor 2 *(*IGF2*) gene, coding a well-characterized growth factor active throughout embryogenesis and fetal growth [2,3]. In normal human tissues only the paternal *IGF2 *allele is transcribed; and its imprinting is regulated by at least two differentially methylated regions (DMRs): one is located upstream of the three *IGF2 *promoters that are subject to imprinting (*IGF2 *DMR), and the other is located upstream of the neighboring non-coding *H19 *gene (*H19 *DMR). The latter region is part of the imprinting control region (ICR) which harbors binding sites for the zinc finger protein CTCF. During early development, imprint marks are erased in the primordial germ cells and new methylation imprints are established according to the germ cell. This has particularly been demonstrated at the *IGF2 *DMR [4] and *H19 *DMR [4,5] in spermatogenic human cell stages. The progressive imprint re-establishment of the DNA methylation imprint marks throughout human spermatogenesis leads to fully methylated *IGF2 *and *H19 *DMRs. Consequently, methylation is only present on the paternally inherited allele in the offspring. Shifts in methylation established at these DMRs can lead to loss of imprinting and transcription of *IGF2 *may be altered [6,7]. Hence, normal physiological mechanisms or homeostasis in the body may be skewed and lead to chronic diseases later in life. Until now, epidemiological studies have focused on maternal factors and especially *in utero *exposures to certain nutritional or environmental conditions as the potential explanation for such disruptions or shifts in methylation at the DMRs [8-10]. This can potentially contribute to a higher risk for obesity [11], chronic diseases at later age [12], including diabetes or cardiovascular diseases [13-15], or even cancer [16,17]. Animal experiments show that modification of maternal diet during development can influence metabolism in adulthood [18]. Although the underlying mechanism and crucial time points of exposure are not clear, changes in epigenetic regulation are now regarded as a highly plausible explanation for linking the associations between dietary exposures in early life to the onset of chronic diseases during adulthood. Several lines of evidence suggest that pre- or periconceptional obesity of the mother may affect metabolic programming [19-21]. Although paternal obesity is equally prevalent, the majority of the epidemiological studies to date suggest *in utero *exposures as the only possible origin of potential epigenomic modifications at birth. Obesity is associated with over-nutrition, unbalanced food intake (such as low vegetable consumption), and a sedentary lifestyle [22]. Consequently, elucidating the epigenetic risks associated with the current "Western" lifestyle on the next generations is crucial. In the current report we determined whether preconceptional obesity, in the mother or the father, is associated with methylation patterns at the *IGF2 *DMRs in newborns using DNA from leukocytes isolated from umbilical cord blood at birth. By including paternal obesity in our study we were able to examine a potential preconceptional impact of the environment on imprint mark reprogramming during male gametogenesis. Consequently, we found that paternal obesity is associated with a decrease in DNA methylation at the *IGF2 *DMR.

## Methods

### Study participants

We studied a subgroup of the first 98 families enrolled in the Newborn Epigenetics Study cohort. Seventy-eight mothers provided detailed information about the biological fathers. One mother gave birth to twins, bringing the total number of newborns with corresponding paternal data to 79. The enrollment process was assisted by a trained interviewer. Detailed recruitment strategies for NEST have been described in a previous study [23]. English speaking pregnant mothers delivered between July 2005 and November 2006 at Duke University Hospital, Durham, NC.

### Data collection

The questionnaire included maternal socio-demographic data, such as age, marital status, race and education, as well as multiple lifestyle or health characteristics (for example, smoking, chronic diseases). One of the main items of the survey included a detailed list of questions about the mother's and the father's height, highest and lowest weight ever, current and usual weight. Medical records were used to verify medical conditions, and to abstract gestation time, birth weight and the newborn's gender. Body mass index was calculated from the data obtained from height and the mother's weight before pregnancy or the father's current weight. Obesity was defined as BMI ≥30 kg/m^2^.

### Specimen collection

At delivery, umbilical cord blood samples were collected in a vacuum blood collection tube coated with K_3_EDTA. The tubes were centrifuged to isolate the buffy coat; this leukocyte-containing buffy coat was then stored at -80^º^C. Genomic DNA was extracted using Gentra Puregene Reagents (Qiagen, Valencia, CA, USA).

### DNA methylation analysis

*IGF2 *and *H19 *DMRs were analyzed by pyrosequencing. The *IGF2 *DMR includes three CpG dinucleotides upstream of *IGF2 *exon 3 (chr 11p15.5; CpG site 1: 2,169,518; CpG site 2: 2,169,515; and CpG site 3: 2,169,499; NCBI Human Genome Build 37/hg19). This DMR has been previously evaluated by Cui *et al*. [6] and Heijmans *et al*. [8]. The region studied for the *H19 *DMR encompasses four dinucleotides located upstream of the *H19 *gene (chr 11p15.5; CpG site 1: 2,024,261, CpG site 2: 2,024,259, CpG site 3: 2,024,257, and CpG site 4: 2,024,254; NCBI Human Genome Build 37/hg19), which is the first of six known sequence motifs that bind the CTCF zinc finger protein [24,25]. The structural characteristics of these *IGF2 *and *H19 *loci and the genomic coordinates of the assays have been presented elsewhere [7,26]. Genomic DNA (800 ng) was treated with sodium bisulfite [27], the *IGF2/H19 *regions were amplified by PCR, and pyrosequencing was performed in duplicate using a Pyromark Q96 MD pyrosequencing instrument (Qiagen). Control assays were also run to validate our methylation results. Detailed methodology, including assay conditions and validation studies, have been described previously [25].

### Statistical methods

**Figure 1:**
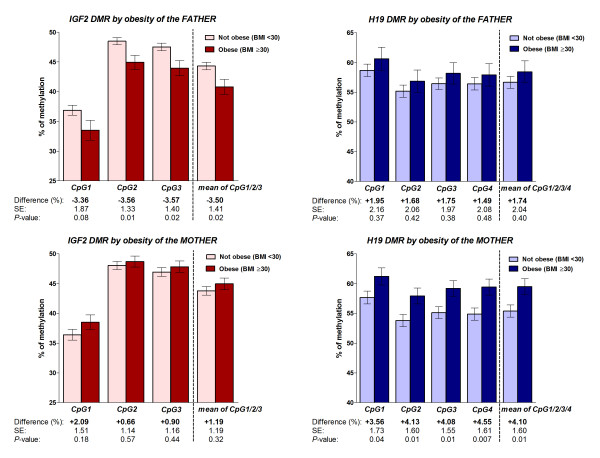
**Methylation at the *IGF2 *and *H19 *DMRs in the offspring by parental obesity**. The graphs represent the mean estimated methylation values of 69 newborns at the *IGF2 *DMR, and 70 newborns at the *H19 *DMR. The *IGF2 *DMR results are based on 14 obese fathers and 25 obese mothers; the results at the *H19 *DMR are based on 15 obese fathers and 23 obese mothers. For each exposure the differences of the least square means of methylation percentages are shown at each CpG site (bold), as well as standard errors (SE) and *P*-values. Bars represent standard errors.

**Figure 2:**
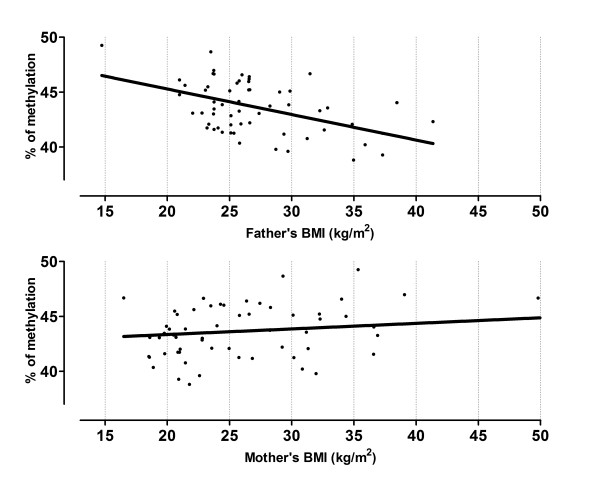
**Offspring's mean methylation % at the *IGF2 *DMR by BMI of the parents**. The predicted methylation means at *IGF2 *DMR are plotted by BMI of the father (upper graph), and BMI of the mother (lower graph); adjusted for maternal age, smoking status, BMI of the other parent, the newborn's birth weight and gender.

Variables were defined as follows: race (African American, Caucasian, and other), age (as a continuous variable), marital status (living with partner *versus *single), at least a college graduate (yes or no), pre-pregnancy maternal and paternal obesity (<30 *versus *≥30 kg/m^2^), smoking status (never/quit smoking when pregnant/continued smoking during pregnancy), birth weight of the baby (<2.5 kg/between 2.5 kg and 3.5 kg/≥3.5 kg), gestation time (<37 weeks *versus *≥37 weeks), and gender of the baby (male or female) (Table 1). Chi Square tests were used to compare obesity of the mother and the father within different sub-groups of pregnant women. If numbers were small (<5) the Fisher exact test was used. We also compared the characteristics for missing and non-missing experimental methylation results and for missing and non-missing BMI of the father. Methylation levels were distributed normally in the groups studied (verified by using the Kolmogorov-Smirnov test). Student's *t*-tests were computed to test for significant differences in the methylation means. We assessed the effect of maternal and paternal obesity (BMI ≥30 kg/m^2^) on the methylation levels of the *IGF2 *and *H19 *DMRs, which were analyzed separately by individual CpG site, as well as by the mean of the CpGs. To account for batch effects from the laboratory tests on different days or different plates we calculated the least square means (or estimated marginal means) of each CpG site. We evaluated potential confounding for paternal and maternal obesity by means of multivariable regression analyses. We used multiple regression analyses, separately for each exposure and characteristics from Table 1. These multiple regression analyses were used to predict DNA methylation at each DMR site. To further explore whether the association between obesity and DNA methylation varied by race, we repeated these analyses in African Americans and Caucasians. Graphs representing the associations between BMI and methylation outcomes were based on the predicted outcomes of the methylation means, after adjusting for maternal age, smoking status, birth weight and gender. Observations included in the regression analyses were determined by the availability of laboratory measures for the *IGF2 *and *H19 *DMRs. All statistical analyses were conducted in SAS v9.2 (SAS Institute Inc., Cary, NC, USA), and GraphPad Prism 5 was used to obtain Figures 1 and 2 (GraphPad Software Inc., La Jolla, CA, USA).

**Table 1:** Parental and newborn characteristics

NEST - Newborn Epigenetics Study Cohort 2005 to 2006		n	%
**BMI mother:**	BMI <30 (not obese)	**59**	**67.8**
	BMI ≥30 (obese)	**28**	**32.2**
**BMI father:**	BMI <30 (not obese)	**63**	**79.7**
	BMI ≥30 (obese)	**16**	**20.3**
**Marital status:**	Living with partner	**72**	**74.2**
	Single	**25**	**25.8**
**Education:**	Low (no college degree)	**57**	**58.8**
	High (at least college degree)	**40**	**41.2**
**Race:**	African American	**38**	**38.8**
	Caucasian	**56**	**57.1**
	Other or not specified	**4**	**4.08**
**Maternal age:**	<30 years	**56**	**57.1**
	≥30 years	**42**	**42.9**
**Smoking:**	Mother never smoked	**45**	**48.4**
	Quit smoking when pregnant	**26**	**27.9**
	Smoked during pregnancy	**22**	**23.7**
**Gestation time:**	Preterm (<37 weeks)	**10**	**10.3**
	Normal (≥37 weeks)	**87**	**86.7**
**Birth weight:**	<2.5 kg	**16**	**16.5**
	≥2.5 kg	**81**	**83.5**
**Baby gender:**	Male	**48**	**49.5**
	Female	**49**	**50.5**

This sub-cohort includes all NEST families from whom babies were born at Duke University Hospital between July 2005 and November 2006. Characteristics of mothers, fathers and newborns are shown for the 98 participants when data were not missing.

### Ethics

The experimental research in this report was performed with the approval of the Duke University Institutional Review Board, reference number: Pro00014548; this means that the research carried out is in compliance with the Helsinki Declaration. All participants signed the informed consent for the use of their medical record data, questionnaire data, their newborn's birth parameters and umbilical cord blood.

## Results

### Characteristics of study participants

The distributions of socio-demographic data are shown in Table 1. Seventy-four percent of the mothers were living with a partner, and nearly 26% were single. The majority of the study population were Caucasians (57.1%) or African Americans (38.8%), and the other races/ethnicities included Asians, Native Americans or non-specified race (4.1%). A quarter of the mothers were between 25 and 29 years old (26.5%). Nearly 59% never obtained a college degree, and were categorized as "low education". Approximately 24% of the mothers reported smoking during pregnancy, and 32% were obese before pregnancy. About 80% of the mothers provided data about the father's characteristics: 20% are obese. Approximately 16% of the newborns had a low birth weight, 10% was born preterm and gender was equally distributed. None of the included characteristics were associated with paternal obesity, with the exception of obesity in the mother (*P *= 0.01). For instance, potential associations between birth weight and parental obesity were excluded by Chi Square tests or Fisher exact tests; *P*-values were 0.7 for paternal and maternal obesity. Maternal obesity was positively associated with single motherhood (*P *= 0.002), being African American (*P *<0.001) and being a non-smoker (*P *= 0.06). Given that 19% of our subjects did not report detailed information about the fathers, we compared all measured characteristics and methylation end-results in newborns with missing anthropometric data about fathers *versus *those without missing data. The majority of the missing paternal data (78%) were from African American mothers with lower education. Single mothers were less likely to answer questions about the father's weight and height (32.0%), compared with married mothers or those living with a partner (13.9%), *P *= 0.07. Other characteristics and the methylation outcomes did not differ significantly between both groups.

### Associations between paternal or maternal obesity and methylation profiles at the *IGF2 *and *H19 *DMRs in the offspring

Using DNA extracted from umbilical cord blood leukocytes we determined the levels of methylation at CpG sites within the *IGF2 *and *H19 *DMRs in newborns. From the 79 samples in our cohort, we obtained experimental data for 67 participants at the *IGF2 *DMR and for 70 participants at the *H19 *DMR. We further refer to these participants as our analytical cohort. No significant differences in characteristics were found when comparing the missing with the non-missing experimental methylation results for both DMRs. We analyzed methylation outcomes by the characteristics described in Table 1 and with the exception of obesity (BMI ≥30 kg/m^2^), none showed significant associations with methylation percentages at the *IGF2 *or *H19 *DMRs in our cohort. Figure 1 shows the estimated means of methylation percentages by obesity of the parents. Differences in methylation percentages among offspring from obese fathers compared to non-obese fathers at CpG1, CpG2, and CpG3 of the *IGF2 *DMR were: -3.36% (*P *= 0.08), -3.56% (*P *= 0.01) and -3.57% (*P *= 0.02), respectively; the mean methylation difference was: -3.50% (*P *= 0.02) (Figure 1). Methylation in offspring among obese fathers was significantly lower than when compared to fathers who were not obese. We observed no differences in methylation percentages at the *IGF2 *DMR between offspring of obese and non-obese mothers. Differences in methylation percentages at CpG1, CpG2 and CpG3 were +2.09% (*P *= 0.18), +0.66% (*P *= 0.57) and +0.90% (*P *= 0.44), respectively; the mean methylation difference was +1.19% (*P *= 0.32) (Figure 1).

At the *H19 *DMR we found no methylation differences in the offspring of obese compared to non-obese fathers. Differences in methylation percentages at CpG1, CpG2, CpG3 and CpG4 of the *H19 *DMR were +1.95% (*P *= 0.37), +1.68% (*P *= 0.42), +1.75% (*P *= 0.38) and +1.49% (*P *= 0.48), respectively; the mean methylation difference was +1.74% (*P *= 0.40) (Figure 1). However, the methylation percentages at the CpG sites of the *H19 *DMR among offspring from obese mothers were significantly higher at CpG1, CpG2, CpG3 and CpG4 compared to offspring from non-obese mothers. The differences were +3.56% (*P *= 0.04), +4.13% (*P *= 0.01), +4.08% (*P *= 0.01) and +4.55% (*P *= 0.007), respectively; the mean methylation difference was +4.10% (*P *= 0.01) (Figure 1). In brief, without adjusting for potential confounders we detected significantly lower methylation at the *IGF2 *DMR associated with paternal obesity, and significantly higher methylation at the *H19 *DMR when the mother was obese.

To assess the independent effects of preconceptional maternal or paternal obesity on DNA methylation at the *IGF2 *and *H19 *DMRs in newborns, we used multiple regression models, controlling for potential confounders, including maternal age, maternal smoking status, gender of the baby, birth weight and experimental batch effects. The results of the regression analyses, found in Table 2, show that paternal obesity is inversely associated with DNA methylation levels at the *IGF2 *DMR in newborns. Controlling for potential confounding did not change this relationship. After adjusting for several characteristics, we observed a significant decrease in methylation when fathers were obese, β-coefficient was -5.28 (*P *= 0.003) (Table 2, Model 3). Little difference for this association was observed when comparing the individual CpG sites. The β-coefficients for CpG1, CpG2 and CpG3 were -5.78 (*P *= 0.01), -5.18 (*P *= 0.002) and -4.76 (*P *= 0.007), respectively. In contrast, results for maternal obesity indicated an increase in methylation at the *IGF2 *DMR, the β-coefficient was +3.08 (*P *= 0.05) when controlling for the same characteristics (Table 2, Model 3). The β-coefficients at the individual CpG-sites were +4.23 (*P *= 0.04), +2.44 (*P *= 0.08), +2.42 (*P *= 0.12), for CpG1, CpG2 and CpG3; with only CpG1 reaching statistical significance.

**Table 2:** Linear Regression Models: methylation at the *IGF2 *and *H19 *DMRs in relation to parental obesity

Linear regression models		*IGF2 *DMR			*H19 *DMR		
	**Obesity of:**	**β**	SE	***P***	**β**	SE	***P***
**Model 1**	Father	**-3.83**	1.48	0.01	**+3.09**	1.64	0.07
**Model 2**	Mother	**+2.38**	1.30	0.08	**+2.80**	1.38	0.05
**Model 3**	Father Mother	**-5.28** **+3.08**	1.62 1.48	0.003 0.05	**+2.55** **+1.05**	1.82 1.70	0.17 0.54

Obesity was defined as BMI ≥30 kg/m^2^. All models were adjusted for maternal age and smoking status, as well as by the newborn's birth weight and gender. Models 1 and 2 include either maternal or paternal obesity. Model 3 includes both maternal and paternal obesity.

In our separate regression models by maternal and paternal obesity, we observed a positive trend in DNA methylation at the *H19 *DMR when adjusting for maternal and newborn characteristics (Table 2, Models 1 and 2, respectively). The β-coefficient was +3.09 when the fathers were obese, but this result was not significant (*P *= 0.07), and +2.80 when the mothers were obese (*P *= 0.05). The multivariate model with the mean methylation percent as a dependent variable and several measured maternal, paternal and newborn characteristics as independent variables (Model 3) showed a β-coefficient for offspring from obese fathers of +2.55 (*P *= 0.17), and a β-coefficient for offspring from obese mothers of +1.05 (*P *= 0.54). Similar results were seen when we evaluated the individual CpG sites (data not shown).

We further stratified our analytical cohort by race (Caucasian and African American) and repeated all regression analyses described above. At the *IGF2 *DMR, the outcome was similar as in our complete analytical cohort. We calculated a β-coefficient of -5.16 in offspring from obese fathers, among Caucasians (*P *= 0.01) (as in Model 3); and a β-coefficient of -3.65 in offspring from obese fathers, among African Americans. Although the estimates suggest an inverse relationship in Caucasian subjects, this effect was not significant among African Americans (*P *= 0.34). These observations were based on only 10 obese fathers in Caucasians, and only 4 obese fathers in African Americans. At the *H19 *DMR, results were also similar and in the same direction as those in our complete analytical cohort. However, we found one higher estimate in Model 1 for children from African American obese fathers: β-coefficient was +8.14 (*P *= 0.007). But, this result was based on a small sample size with only 6 obese African American fathers. Together with our statistical power calculations we conclude that the stratified analyses represent unstable estimates.

We further extended the regression analyses in our analytical cohort by replacing the dichotomous obesity variable by the continuous BMI variable. At the *H19 *DMR, we found a β-coefficient of +0.21 (*P *= 0.12) for paternal BMI and +0.16 (*P *= 0.09) for maternal BMI, controlling for the same variables as before; but these results were still not significant. Whereas at the *IGF2 *DMR, our data reached a significant positive association when both paternal and maternal BMI were used as independent variables in the multivariate model; β-coefficients were -0.38 (*P *= 0.009), and +0.22 (*P *= 0.02), respectively. These correlations between parental BMI and the predicted mean methylation levels at the *IGF2 *DMR in the offspring are depicted in Figure 2.

## Discussion

We explored the potential effect of parental obesity on *IGF2*/*H19 *DMR methylation in newborns. We found a significant decrease in methylation among newborns of obese fathers at the *IGF2 *DMR in DNA extracted from cord blood leucocytes. This finding remained significant after controlling for potential confounders (β-coefficient = -5.28, *P *= 0.003). Hypomethylation at the *IGF2 *DMR has been associated with an increased risk of developing cancers, such as Wilms' tumor [28], colorectal cancer [6] and ovarian cancer [7]. We found no significant changes in methylation levels associated with paternal obesity at the *H19 *DMR region.

Obesity is a metabolic condition that has paradoxically been associated with poverty, low quality of life, malnutrition and an imbalanced intake of nutrients [21,22]. Few epidemiological studies indicate potential correlations between obesity or food supplies in the paternal line and offspring's birth weight [29], body-fat in prepubertal girls [30], or mortality from chronic diseases [31-33]. Epidemiological data regarding associations between maternal obesity and the offspring's birth weight vary by study (reviewed by McDonald *et al*. [34]). We examined possible associations between paternal or maternal obesity and birth weight but detected no associations. Epidemiological studies on maternal obesity-related exposures generally show a positive association between abnormally high BMI and congenital anomalies (reviewed by Stothard *et al*. [35]). These harmful effects are mostly attributed to *in utero *exposures to malnutrition or overnutrition; while very often, data indicate the importance of exposures at the very early stages of development, even before conception. Research in animal models suggests that potential diet-dependent transgenerational effects may be explained by changes in the establishment of epigenetic gene regulatory marks [12,36-38]. Analyses of adults born to mothers exposed to poor nutrition during the Dutch famine indicated a 5% decrease in methylation at the *IGF2 *DMR compared to the same sex non-exposed siblings. Interestingly, the magnitude of this effect is similar to the effect we observe in offspring from obese fathers at the same locus. In the Dutch famine cohort, exposure during the periconceptional period was more important than during the second or the third trimester of gestation [8]. Furthermore, not only do exposures during early gestation cause harmful health outcomes, but famine prior to conception has also been associated with poor health [39]. Similar results were found among Gambian children; they exhibit altered DNA methylation at several metastable epialleles according to the seasonal nutritional circumstances in which they were conceived [40]. Both cohorts described above did not directly examine the relationship with the fathers' dietary patterns; although they were most likely exposed to the same famine or nutritional circumstances as the mothers. Analysis of the Framingham Heart Study indicates that early-onset paternal obesity, and not maternal obesity, increases the odds of aberrant serum levels of the metabolic biomarker ALT (alanine transaminase) in the offspring [41]. Studies on animal models have shown similar associations. In rats, a high fat paternal diet results in offspring with early onset of impaired insulin secretion, altered expression of multiple genes related to normal pancreatic beta-cell function, and altered methylation at a putative regulatory region of the *Interleukin 13 receptor, alpha 2 *gene [42]. Male mice whose mothers were exposed to a high-fat diet were not only obese, insulin insensitive and diabetic, they were also capable of passing part of this phenotype to the next generation, suggesting an underlying epigenetic mechanism transmitted through germ cells [43]. To our knowledge, our study is the first epidemiological study that suggests a similar underlying epigenetic mechanism conferred by harmful paternal dietary patterns or obesity.

Obesity is associated with elevated IGF2 circulating levels [44] and increased estrogen levels [45]. Although we did not include IGF2 protein levels in parents or offspring in our current analyses, we earlier showed that hypomethylation at the *IGF2 *DMR is associated with higher circulating IGF2 levels in the offspring [25,46]. This association was strongest in offspring from obese mothers, independent of race. In brief, a decrease of 5% at the *IGF2 *DMR methylation corresponded to an increase of at least 10% in serum concentration of IGF2 [46]. In addition, other studies have shown that small aberrant methylation changes at the *IGF2 *or *H19 *DMR is linked to increased expression of IGF2 [6,7,24,25,47], as well as an increased susceptibility to chronic diseases [48-51]. Similar small effects on DNA methylation have also been associated with the use of assisted reproductive technologies [52], the use of psychotropic drugs during pregnancy [53] and smoking [25]. These subtle epigenetic changes have been described as adaptive responses to the environment, while major epigenetic shifts during development would cause more detrimental consequences [25]. Furthermore, exploring the CpG sites at the *IGF2 *and *H19 *DMRs may represent only a fraction of changes occurring elsewhere in the genome. Environmental factors, among which is diet, have been associated with changes in DNA methylation and may have profound effects on genomic imprinting; accumulation of these effects may result in disturbed metabolic homeostasis [54]. Follow-up studies on the anthropometric and other developmental factors of the NEST children are underway to further examine the influence of small changes in DNA methylations at several DMRs on childhood obesity or other adverse consequences. Evidence in animal studies indicates that DNA methylation at the *IGF2/H19 *locus in sperm might be under tight control of estrogen [55], produced by adipocytes. This suggests a mechanism by which increased exposure to estrogen could lead to inadequate establishment of methylation at the *IGF2 *DMR in sperm. Alternatively, obesity-related factors may also disrupt functioning of other components of the epigenetic machinery leading to an inability to appropriately establish imprint marks during spermatogenesis, which is ongoing through adult male life [5]. Offspring of obese fathers may, therefore, demonstrate incomplete methylation. In order to further explore these hypotheses, more research on the epigenetic effects of obesity on human germ cells is needed.

Although our bivariate analysis did not indicate an association between maternal obesity and DNA methylation at the *IGF2 *DMR in newborns, the regression analysis showed that controlling for paternal obesity resulted in an opposite effect, meaning that while paternal obesity was associated with a decrease in methylation, maternal obesity tended to be associated with an increase in methylation, but this was only significant at one CpG site. Using pre-pregnancy BMI instead of obesity in our multivariate analysis strengthened this association. At the *H19 *DMR, the bivariate analysis showed an increase of 4.1% when mothers were obese (*P *= 0.01). An increase in DNA methylation by maternal pre-gestational BMI has also been reported earlier in cord blood samples, more particularly at the PPARG promoter [56]. Hypermethylation at the *IGF2 *or *H19 *DMR has been associated with loss of imprinting of *IGF2*, and several disorders. For instance, *IGF2 *imprinting defects have been implicated in Silver-Russell syndrome [57], Wilms' tumor [24,58], hepatoblastoma [59] and ovarian cancer [7]. Our regression analyses at the *H19 *DMR showed that adjusting for potential confounders, including paternal obesity or BMI, diminished this positive association (Table 2, Model 3). We attribute this to the fact that maternal and paternal obesity are closely related and the fact that methylation outcomes for both parental exposures are in the same direction. A larger study is necessary to further explore the potential impact of parental obesity on DNA methylation at the *H19 *DMR. Furthermore, the exposure from oocyte stage till birth to maternal obesity or related lifestyles is complex. Hormonal factors that may influence DNA methylation cannot be ruled out. It has been shown that the rat *H19 *DMR has an estrogen responsive element, suggesting that estrogen can form a complex with Dnmt1, a DNA methyl transferase, leading to DNA methylation at a normally unmethylated maternal allele [55].

A potential limitation of our study includes the use of cord blood as a marker for the newborn's epigenetic status. However, we used isolated leucocytes and *IGF2 *is a well studied imprinted gene whose germline DMRs should be similarly methylated across all cell types, given the establishment of the epigenetic profile prior to conception. *IGF2 *and *H19 *DMR methylation profiles were verified in DNA from different cell fractions from umbilical cord blood and we found no differences across the cell types [26]. Another possible limitation is proof of paternity, and the reliability of the paternal anthropometric data, which were reported by pregnant mothers. However, the questions regarding the anthropometric data were detailed and verified for consistency. We do not expect that methylation outcomes are differential with respect to the potential misclassification of exposures. Nineteen percent of our population had missing data about the father. The methylation outcomes of these newborns were not included in our final analytical study group. However, we compared methylation outcomes by missing and non-missing characteristics of the fathers and found no differences. We also compared all measured maternal and newborn characteristics in both groups and most characteristics were similarly distributed. We found no significant differences when mothers were single or not, but found significant differences by education and race. Most missing data were among the lower educated and African American mothers. As far as we could test, education was not associated with obesity in either of the parents, and race was only associated with obesity of the mother. It is very likely we missed a number of obese fathers from African Americans. We cannot verify if paternal obesity is equally distributed in all subgroups of missing and non-missing paternal data, and, therefore, selection bias cannot be excluded. However, when reanalyzing our regression models with race included as an independent variable, our results remained the same; only the effect of maternal obesity attenuated in Model 2 (Table 2) at *H19 *DMR, the β-coefficient for maternal obesity became +2.18 (SE = 1.65, *P *= 0.19) (data not shown). Possibly, race is on the same causal pathway as obesity, regarding the effect on methylation outcomes. As mentioned in the results section, race by itself was not associated with methylation outcomes at neither of the two DMRs studied in this cohort. Nevertheless, given our earlier analyses on maternal exposures showed a race dependent effect at the same imprinted loci [53], we further stratified our data by race. The outcomes were in the same direction as the complete analytical cohort, but given the smaller numbers, the results represented unstable estimates. In general, the small sample size remains a limitation in our study which may partly explain why our results at the *H19 *DMR do not reach statistical significance. Nevertheless, the data concerning associations between *IGF2 *DMR methylation and parental BMI or obesity reached sufficient power, especially when studying offspring from fathers with high BMI.

## Conclusions

Our early findings suggest that lifestyle factors of parents may be indirectly transmitted to the next generation via epigenetic mechanisms. We show that one of the measurable lifestyle parameters, obesity of the father, is associated with hypomethylation at the *IGF2 *DMR in the offspring. Aberrant low methylation at this DMR has been associated with adverse health outcomes. As a consequence, our preliminary result may be important for public health, especially in regard to the global burden of obesity. We hypothesize that the molecular mechanism behind our observation might be a hormonal difference between obese and non-obese parents, inducing an incomplete or unstable establishment of methylation at the *IGF2 *DMR during gametogenesis. As a result, exposures to adverse lifestyle factors or poor/over-nutrition during spermatogenesis may affect the reprogramming of methylation profiles at imprinted genes. Further research is necessary to confirm this hypothesis. Although our study population is small, this is the first report in humans suggesting that paternal obesity may disrupt the normal establishment of genomic imprinting in germ cells.

## Abbreviations

BMI: body mass index; DMRs: differentially methylated regions; ICR: imprinting control region; *IGF2*: Insulin-like Growth Factor 2; NEST: Newborn Epigenetics Study

## Competing interests

The authors declare that they have no competing interests.

## Authors' contributions

AS developed the hypothesis of this study, designed the analytical strategy, analyzed the data and wrote the manuscript. JS contributed to the analysis and interpretation of the data, and helped to draft the manuscript. AM oversaw participant recruitment in the clinic and contributed to editing the manuscript. FW implemented the statistical analysis. ZH performed the assays. AB contributed to the research discussions and the editing of the manuscript. JK contributed to the logistics of data collection. RJ contributed to the inception of the original NEST research hypothesis. SM is co-principal investigator and oversaw laboratory analysis and processing of the specimens and helped to draft the manuscript. CH is the principle investigator who oversaw the design and conduct of NEST. All authors have read the manuscript and given their final approval of submission for publication.

## Pre-publication history

The pre-publication history for this paper can be accessed here:

http://www.biomedcentral.com/1741-7015/11/29/prepub
